# Treatment of hereditary angioedema—single or multiple pathways to the rescue

**DOI:** 10.3389/falgy.2022.952233

**Published:** 2022-09-12

**Authors:** Anna Valerieva, Hilary J. Longhurst

**Affiliations:** ^1^ Department of Allergology, Medical University of Sofia, Sofia, Bulgaria; ^2^ Department of Immunology, Auckland District Health Board, and Department of Medicine, University of Auckland, Auckland, New Zealand

**Keywords:** bradykinin B_2_ receptor antagonist, complement C1 inhibitor protein, hereditary angioedema, kallikreins, prophylaxis

## Abstract

Hereditary angioedema (HAE) is a rare disease caused by mutations in the *SERPING1* gene. This results in deficient or dysfunctional C1 esterase inhibitor (C1-INH) and affects multiple proteases involved in the complement, contact-system, coagulation, and fibrinolytic pathways. Current options for the treatment and prevention of HAE attacks include treating all affected pathways *via* direct C1-INH replacement therapy; or specifically targeting components of the contact activation system, in particular by blocking the bradykinin B_2_ receptor (B2R) or inhibiting plasma kallikrein, to prevent bradykinin generation. Intravenously administered plasma-derived C1-INH (pdC1-INH) and recombinant human C1-INH have demonstrated efficacy and safety for treatment of HAE attacks, although time to onset of symptom relief varied among trials, specific agents, and dosing regimens. Data from retrospective and observational analyses support that short-term prophylaxis with intravenous C1-INH products can help prevent HAE attacks in patients undergoing medical or dental procedures. Long-term prophylaxis with intravenous or subcutaneous pdC1-INH significantly decreased the HAE attack rate vs. placebo, although breakthrough attacks were observed. Pathway-specific therapies for the management of HAE include the B2R antagonist icatibant and plasma kallikrein inhibitors ecallantide, lanadelumab, and berotralstat. Icatibant, administered for treatment of angioedema attacks, reduced B2R-mediated vascular permeability and, compared with placebo, reduced the time to initial symptom improvement. Plasma kallikrein inhibitors, such as ecallantide, block the binding site of kallikrein to prevent cleavage of high molecular weight kininogen and subsequent bradykinin generation. Ecallantide was shown to be efficacious for HAE attacks and is licensed for this indication in the United States, but the labeling recommends that only health care providers administer treatment because of the risk of anaphylaxis. In addition to C1-INH replacement therapy, the plasma kallikrein inhibitors lanadelumab and berotralstat are recommended as first-line options for long-term prophylaxis and have demonstrated marked reductions in HAE attack rates. Investigational therapies, including the activated factor XII inhibitor garadacimab and an antisense oligonucleotide targeting plasma prekallikrein messenger RNA (donidalorsen), have shown promise as long-term prophylaxis. Given the requirement of lifelong management for HAE, further research is needed to determine how best to individualize optimal treatments for each patient.

## Introduction

Hereditary angioedema (HAE) is a rare and potentially life-threatening genetic disease that can cause recurrent episodes (attacks) of nonpruritic swelling of the skin, affecting the extremities (e.g., hands, feet), face, and genitals, as well as submucosal swelling of the gastrointestinal and upper respiratory tracts (1–3). Approximately 85% of patients have type I HAE (type I C1-INH-HAE), which is characterized by a deficiency in C1 esterase inhibitor (C1-INH) levels (2–4). Type II HAE (type II C1-INH-HAE) accounts for about 15% of cases and is associated with abnormal function of C1-INH in the presence of normal C1-INH levels. These 2 types of HAE are caused by mutations in the *SERPING1* gene, which encodes C1-INH (2). A third type of HAE, in which both levels and function of C1-INH are normal (HAE-nl-C1INH) (2, 5), is associated with specific genetic mutations (i.e., *F12*, *ANGPT1*, *HS3ST6*, *PLG*, *MYOF*, and *KNG1*), although many patients with HAE-nl-C1INH have no currently identified genetic mutation (2, 6, 7). HAE substantially impairs patient health-related quality of life, disrupts daily activities, and adversely affects social and professional and/or academic functioning (3, 8–11). Additionally, HAE is associated with increased rates of anxiety and depression, likely related to the unpredictability of symptoms and the associated emotional and physical stress (8–11). Severe HAE attacks require immediate intervention and may necessitate an emergency department visit or hospitalization, adding to the disease burden (11, 12).

The ultimate goal of HAE treatment is to achieve complete disease control (i.e., prevent all HAE attacks) and normalize patients’ quality of life and daily functioning (12). For those patients who are unable to achieve complete disease control, therapy aims to reduce the number of HAE attacks and improve quality of life. Pharmacologic management of HAE consists of on-demand (acute) treatment of HAE attacks, short-term prophylaxis delivered prior to procedures or events anticipated to trigger HAE symptoms, and routine, long-term prophylaxis to prevent HAE attacks (2, 13). On-demand therapy aims to minimize morbidity and prevent mortality during an HAE attack, while long-term prophylaxis is broadly deemed valuable for helping to optimize patients’ quality of life and daily functioning. Minimizing the burden of treatment and associated adverse effects is also an important consideration, especially in patients receiving long-term prophylaxis (12, 13). In recent years, the range of pharmacologic treatment options for the management of HAE has expanded considerably to encompass agents with more convenient routes of administration that facilitate self-management, as well as medications with different mechanisms of action that target distinct components of the pathways involved in HAE (14). Multiple treatment options offer the opportunity to individualize therapy, provide an opportunity for both prophylaxis and on-demand therapy using synergistic mechanisms of action, and minimize the disease burden. This narrative review describes the biologic pathways of importance in HAE and provides an overview of therapies that target these pathways to prevent and treat HAE attacks.

## Pathways of importance in HAE

**Figure 1:**
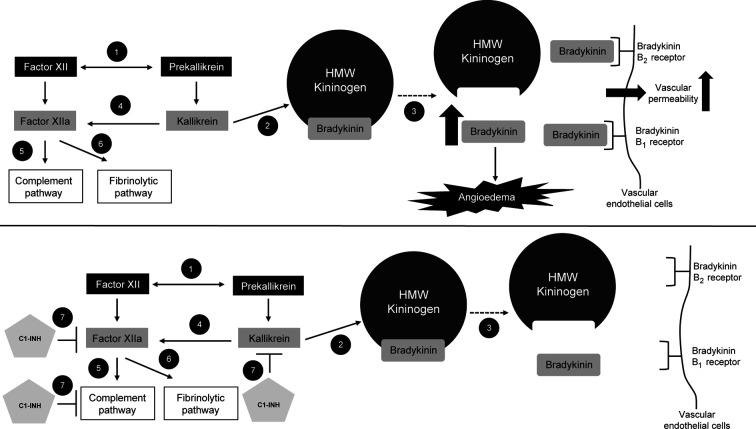
Dysregulation of signaling pathways in HAE. (1) When activated by trace amounts of factor XIIa, plasma prekallikrein and factor XII cleave each other to generate kallikrein and factor XIIa. (2) Kallikrein cleaves HMW plasma kininogen, leading to (3) the release of bradykinin. (4) Plasma kallikrein cleaves factor XIIa, leading to (5) activation of complement and (6) fibrinolytic pathways. In the top figure, the increase in bradykinin levels results in angioedema. Bradykinin binds bradykinin B_1_ and B_2_ receptors on vascular endothelial cells, leading to an increase in vascular permeability. In the bottom figure, (7) C1-INH inhibits factor XIIa, the complement pathway, and kallikrein, thus leading to a decrease in bradykinin production and reduced activation of bradykinin B_1_ and B_2_ receptors on vascular endothelial cells. *C1-INH, C1 esterase inhibitor; HAE, hereditary angioedema; HMW, high molecular weight*. *Figure created with data from Cicardi M, et al. J Allergy Clin Immunol Pract. (2018) 6(4):1132–41; and Zuraw BL. N Engl J Med. (2008) 359(10):1027–36* (5, 16).

The plasma contact system is composed of the enzymes factor XII and plasma prekallikrein and is involved in the generation of the inflammatory peptide bradykinin and in blood coagulation (15). This system, together with the nonenzymatic co-factor high molecular weight (HMW) kininogen, comprises the kallikrein–kinin pathway (15, 16). Factor XII is activated by a number of mechanisms to generate factor XIIa (activated factor XII), which then cleaves kallikrein from prekallikrein. Kallikrein further activates factor XII in a positive feedback loop and also cleaves the HMW kininogen, releasing bradykinin (Figure 1) (5, 16). Bradykinin plays a role in blood coagulation, fibrinolysis, and vasodilation and is the primary mediator of enhanced vascular permeability during an HAE attack (5, 15, 16). Additionally, cleavage of factor XIIa by kallikrein results in release of the active protease factor XIIf, which activates the classical complement pathway (16).

Under healthy conditions, C1-INH inhibits several proteases, including factors XIIa and XIIf and plasma kallikrein, as well as components of the early classical complement pathway (Figure 1) (5, 16). In types I and II HAE, C1-INH deficiency or dysfunction causes an increase in bradykinin levels because of dysregulation of the plasma contact system (5, 16). Unabated generation of bradykinin, resulting from insufficient C1-INH regulation of factor XIIa and kallikrein, leads to angioedema. Plasma kallikrein cleaves factor XIIa, leading to activation of the complement cascade; activation of complement results in the cleavage of C5 (to the anaphylatoxin C5a) and formation of the complement fragment C4a, which increases endothelial permeability (17). Cytokines can also affect endothelial permeability, with proinflammatory cytokines (e.g., interleukin [IL]-1, IL-4, IL-6, IL-8, IL-13) increasing permeability and anti-inflammatory cytokines (e.g., IL-1Ra, IL-10) inhibiting permeability (17). While evidence supports a key role of the bradykinin B_2_ (B2) receptor in angioedema, upregulation of the bradykinin B_1_ (B1) receptor during stress, trauma, or infection may influence susceptibility to angioedema, and prolonged B1 signaling may be involved in sustaining swelling during an HAE attack (16, 18). Thus, it is unclear whether blockade of both B1 and B2 receptors may be needed to completely prevent vascular leakage and associated swelling (18).

The lectin pathway is an activator of the complement cascade, which is mediated, in part, through mannose-binding lectin–associated serine proteases (MASPs) (19). MASP-1 is able to directly cleave HMW kininogen to generate and release bradykinin. C1-INH is an important regulator of the lectin pathway *via* inhibition of MASP-1 and MASP-2. Thus, dysregulation of MASPs may play a role in the pathophysiology of HAE by contributing to elevated bradykinin levels. The fibrinolytic pathway is also activated by factor XIIa (Figure 1), leading to conversion of plasminogen to plasmin; plasmin subsequently cleaves fibrin, leading to fibrin degradation (5). This pathway plays a greater role in some types of HAE-nl-C1INH pathophysiology, but also contributes to the endothelial dysfunction of types I and II HAE (20).

Pathways important in C1-inhibitor deficiency in HAE may also play a role in autoimmunity and neoplasms. A Swedish population-based cohort study reported that patients with HAE were at increased risk of autoimmune disease compared with individuals in the general population (odds ratio [OR], 1.6; 95% confidence interval [CI], 1.2–2.4); the risk of developing systemic lupus erythematosus was significantly greater in patients with HAE compared with individuals without HAE (OR, 71.9; 95% CI, 8.8–586.7) (21). While this study did not find an increased risk of cancer in patients with HAE vs. the general population (OR, 0.9; 95% CI, 0.6–1.4) (21), a retrospective review of medical records in Italy reported that neoplasms were the most common cause of mortality in patients with HAE, compared with cardiovascular disease as the most common cause of mortality in the general population (22). However, future studies are warranted to examine the role of HAE pathways in autoimmune disease and cancer.

## Multiple pathway therapies

### C1-INH replacement therapy

C1-INH products provide a direct replacement for the low levels or low functional activity of C1-INH in patients with type I or II HAE, respectively (13). Accordingly, administration of C1-INH replacement therapy during an HAE attack restores regulation of the cascade systems producing bradykinin by inhibiting the same targets as endogenous C1-INH (i.e., plasma kallikrein, factors XIIa and XIIf, and elements of the complement pathway, including MASP-1; Figure 1) (5, 13, 16).

#### On-demand therapy

The World Allergy Organization (WAO)/European Academy of Allergy and Clinical Immunology (EAACI) guidelines recommend treatment with intravenous (IV) C1-INH as soon as possible after onset of an HAE attack (13). Intravenously administered plasma-derived C1-INH (pdC1-INH; Berinert; CSL Behring LLC; Kankakee, IL) and recombinant human C1-INH (rhC1-INH; Ruconest; Pharming Healthcare Inc.; Warren, NJ; Table 1) (23–37) are efficacious and well tolerated as on-demand treatment of HAE attacks (Table 2) (38–41). In a phase 2/3, randomized, double-blind, placebo-controlled trial (*n* = 124), pdC1-INH (Berinert) 20 U/kg provided a significantly faster onset of symptom relief of abdominal or facial attacks compared with placebo (median, 0.5 vs. 1.5 h, respectively; *P *= 0.002) and a significantly shorter median time to complete resolution of symptoms (4.9 vs. 7.8 h, respectively; *P *= 0.02; Table 2) (38). These data were supported by an open-label extension trial (*N* = 57) of pdC1-INH 20 U/kg as on-demand treatment (median follow-up, 24 months; Table 2) (39). Another pdC1-INH product (Cinryze; ViroPharma Biologics LLC; Lexington, MA) administered as on-demand treatment also showed significantly faster onset of symptom relief compared with placebo (40) (Table 2) but is not approved for acute treatment of HAE in the United States or European Union (Table 1).

**Table 1:** Approved treatments for hereditary angioedema.

Treatment	Route of administration	FDA-approved indication and dose	EMA-approved indication(s) and dose	MHLW-approved indication(s) and dose
*C1-INH replacement products*				
pdC1-INH (Berinert)	IV	Acute treatment in pediatric patients and adults (20 IU/kg)	Acute treatment (20 IU/kg) and short-term prophylaxis (pediatric patients: 15–30 IU/kg; adults: 1,000 IU)	Acute treatment and short-term prophylaxis (<50 kg: 500 U; >50 kg, 1,000–1,500 U)
pdC1-INH (Cinryze)	IV	Long-term prophylaxis in patients aged ≥6 y (children 6–11 y: 500 U q3–4 d; adolescents ≥12 y and adults: 1,000 U q3–4 d)	Acute treatment (2–11 y [10–25 kg]: 500 IU; 2–11 y [>25 kg: 1,000 IU]; ≥12 y: 1,000 IU) and short-term prophylaxis in patients aged ≥2 y (2–11 y [10–25 kg]: 500 IU <24 h; 2–11 y [>25 kg]: 1,000 IU <24 h; ≥12 y: 1,000 IU <24 h); long-term prophylaxis in patients aged ≥6 y (6–11 y: 500 IU q3–4 d; ≥12 y: 1,000 IU q3–4 d)	Not approved
rhC1-INH/Conestat alfa (Ruconest)	IV	Acute treatment in adolescents and adults (<84 kg: 50 U/kg; ≥84 kg: 4,200 U)	Acute treatment in patients aged ≥2 y (<84 kg: 50 U/kg; ≥84 kg: 4,200 U)	Not approved
pdC1-INH (Haegarda [US]/Berinert [EU])	SC	Long-term prophylaxis in patients aged ≥6 y (60 IU/kg q3–4 d)	Long-term prophylaxis in adolescents and adults (60 IU/kg)	Not approved
*Bradykinin B2 receptor antagonist*				
Icatibant (Firazyr)	SC	Acute treatment in patients aged ≥18 y (30 mg)	Acute treatment in patients aged ≥2 y (patients 2–17 y: 12–25 kg, 10 mg; 26–40 kg, 15 mg; 41–50 kg, 20 mg; 51–65 kg, 25 mg; >65 kg, 30 mg; adults: 30 mg)	Acute treatment in adults (30 mg)
*Plasma kallikrein inhibitors*				
Ecallantide (Kalbitor) (33)	SC	Acute treatment in patients aged ≥12 y (30 mg)	Not approved	Not approved
Lanadelumab (Takhzyro) (34, 35)	SC	Long-term prophylaxis in patients aged ≥12 y (300 mg q2–4 wk)	Long-term prophylaxis in patients aged ≥12 y (300 mg q2–4 wk)	Long-term prophylaxis in patients aged ≥12 y (300 mg q2–4 wk)
Berotralstat (Orladeyo) (36, 37)	Oral	Long-term prophylaxis in patients aged ≥12 y (150 mg once daily)	Long-term prophylaxis in patients aged ≥12 y (150 mg once daily)	Long-term prophylaxis in patients aged ≥12 y (150 mg once daily)

EMA, European Medicines Agency; FDA, US Food and Drug Administration; IV, intravenous; MHLW, Ministry of Health, Labour and Welfare (Japan); pdC1-INH, plasma-derived C1 esterase inhibitor; SC, subcutaneous.

**Table 2:** Summary of clinical trials of C1-INH replacement therapy as on-demand treatment for HAE attacks.

Therapy	Study and inclusion criteria	Treatments	Efficacy outcome(s)	Safety
pd C1-INH (Berinert)	Craig TJ, et al. (38) IMPACT1 August 2005–December 2007 R, DB, PBO-C, ph 2/3 study Pts ≥6 y with type I or II HAE with single abdominal or facial attacks	pdC1-INH 10 U/kg bw IV (*n* = 39) pdC1-INH 20 U/kg bw (*n* = 43) IV PBO (*n* = 42)	Time to onset of symptom relief^a^: Mean (SD): pdC1-INH 10 U/kg: 7.5 h (10.5) pdC1-INH 20 U/kg: 3.9 h (8.2) PBO: 10.3 h (11.5) Median (range): pdC1-INH 10 U/kg: 1.2 h (0.2–24.0) pdC1-INH 20 U/kg: 0.5 h (0.2–24.0) PBO: 1.5 h (0.2–24.0) *P *= 0.002 (20 U/kg vs. PBO) Time to complete resolution of HAE symptoms: Mean (SD): pdC1-INH 10 U/kg: 216.1 h (494.2) pdC1-INH 20 U/kg: 81.8 h (314.3) PBO: 125.1 h (382.8) Median (range): pdC1-INH 10 U/kg: 20.0 h (0.5–1,486.2) pdC1-INH 20 U/kg: 4.9 h (0.5–1,486.2) PBO: 7.8 h (0.3–1,486.2) *P *= 0.02 (20 U/kg vs. PBO)	pdC1-INH 10 U/kg (*n* = 39) pdC1-INH 20 U/kg (*n* = 46) PBO (*n* = 41) Any AE ≤4 h of start of tx: pdC1-INH 10 U/kg, 25.6% (*n* = 10); pdC1-INH 20 U/kg, 19.6% (*n* = 9) vs. PBO 43.9% (*n* = 18) Tx-related AEs: 20.5% (*n* = 8), 10.9% (*n* = 5), vs. 19.5% (*n* = 8), respectively SAEs/AEs leading to discontinuation: 0 (all groups) Most common AEs^b^: Muscle spasms: 10.3% (*n* = 4), 2.2% (*n* = 1), vs. 4.9% (*n* = 2) Pain: 10.3% (*n* = 4), 2.2% (*n* = 1), vs. 2.4% (*n* = 1) Nausea: 2.6% (*n* = 1), 6.5% (*n* = 3), vs. 12.2% (*n* = 5) Diarrhea: 2.6% (*n* = 1), 0, vs. 9.8% (*n* = 4)
	Craig TJ, et al. (39) (IMPACT2) August 2005–February 2010 OL extension of IMPACT1 Pts ≥6 y with type I or II HAE who previously participated in IMPACT1 (*n* = 57; 1,085 attacks) with attacks at any body location	pdC1-INH 20 U/kg bw IV Median study duration: 24 mo (range, 0–51 mo) Pts received tx for median 7 attacks (range, 1–184 attacks)	Per-attack analysis: Median (range) time to onset of symptom relief^a^: 0.4 h (0.05–497.0^c^) Median (range) time to complete resolution of HAE symptoms: 14.3 h (0.2–497.0^c^) Single dose effective for 1,073/1,085 HAE attacks (99%) Per-pt analysis: Median time to onset of symptom relief^a^: 0.46 h (95% CI, 0.39–0.53; range, 0.2–497.0^c^) Median time to complete resolution of HAE symptoms: 15.5 h (95% CI, 11.6–21.6; range, 0.6–497.0^c^)	AEs by no. of pts (*n* = 57)/no. of attacks (*n* = 1,085): Any AE: 43.9% (*n* = 25)/5.4% (*n* = 59) Tx-related AEs: 14.0% (*n* = 8)/0.8% (*n* = 9) SAEs: 1.8% (*n* = 1)^d^/<0.1% (*n* = 1)^d^ AEs leading to discontinuation: 1.8% (*n* = 1)/<0.1% (*n* = 1) Most common AEs^c^: Headache: 8.8% (*n* = 5)/0.7% (*n* = 8) Nasopharyngitis: 5.3% (*n* = 3)/0.3% (*n* = 3)
pdC1-INH (Cinryze)	Zuraw BL, et al. (40) 14 March 2005–18 May 2007 R, DB, PBO-C Pts ≥6 y with single HAE attacks (abdomen, external genitalia, extremities, face, throat)	pdC1-INH 1,000 U/10 ml (*n* = 36) PBO (*n* = 35)^e^	Median time to onset of symptom relief: pdC1-INH 1,000 U: 2 h PBO: >4 h Estimated success rate ratio, 2.4 (95% CI, 1.2–5.0; *P *= 0.02) Pts with time to relief ≤4 h: 21/35 (60%) vs. 14/33 (42%; *P *= 0.06) Median time to complete resolution of symptoms (after second dose of study drug [pdC1-INH: *n* = 23; PBO: *n* = 28]): 12.3 vs. 25.0 h (*P *= 0.004)	pdC1-INH 1,000 U/10 ml (*n* = 36) PBO (*n* = 35) ≥1 AEs: 17% (*n* = 6) vs. 20% (*n* = 7) Possibly tx-related AEs pdC1-INH: Rash at injection site: 2.8% (*n* = 1) PBO: Contact dermatitis: 2.9% (*n* = 1) Tetany: 2.9% (*n* = 1)
rhC1-INH Ruconest	Zuraw B, et al. (41) R, DB, C 2 independent trials with similar design, entry criteria, endpoints Pts ≥12 y (US/Canada) or ≥16 y (Europe)	rhC1-INH 50 U/kg bw IV^f^ (*n* = 12) rhC1-INH 100 U/kg bw IV (*n* = 29) PBO (*n* = 29)	Time to onset of symptom relief: Median (range): rhC1-INH 50 U/kg: 122 min (72–136) *P *= 0.01 vs. PBO rhC1-INH 100 U/kg: 66 min (61–122) *P *< 0.001 vs. PBO PBO: 495 min (245–520)	rhC1-INH 50 U/kg (*n* = 12) rhC1-INH 100 U/kg (*n* = 29) PBO (*n* = 29) Any AE ≤90 d of tx administration: rhC1-INH 50 U/kg, 33% (*n* = 4); rhC1-INH 100 U/kg, 24% (*n* = 7), vs. 48% (*n* = 14) Tx-related AEs: 0, 3% (*n* = 1), vs. 10% (*n* = 3) SAEs: 0, 3% (*n* = 1), vs. 10% (*n* = 3) AEs leading to discontinuation: 0 (all groups) Most common AEs^b^: Headache: 0, 10% (*n* = 3), vs. 14% (*n* = 4) Back pain: 8% (*n* = 1), 0, vs. 0 CRP: 8% (*n* = 1), 0, vs. 0 Erythema: 8% (*n* = 1), 0, vs. 0 Pruritus: 8% (*n* = 1), 0, vs. 0 Tooth abscess: 8% (*n* = 1), 0, vs. 0 UTI: 8% (*n* = 1), 0, vs. 0

AE, adverse event; bw, body weight; C, controlled; C1-INH, C1 esterase inhibitor; CRP, C-reactive protein; DB, double-blind; HAE, hereditary angioedema; IMPACT, International Multicenter Prospective Angioedema C1-INH Trial; IV, intravenous; OL, open-label; PBO, placebo; PBO-C, placebo-controlled; pdC1-INH, plasma-derived C1 esterase inhibitor; ph, phase; pts, patients; R, randomized; rhC1-INH, recombinant human C1 esterase inhibitor; SAE, serious adverse event; tx, treatment; UTI, urinary tract infection.

^a^ Time from the start of treatment to the onset of symptom relief, as determined by patients’ responses to a standard question posed at appropriate time intervals for as long as 24 h after the start of treatment.

^b^ In any group, the most common AEs.

^c^ One patient was discontinued from the study after receiving treatment for an abdominal attack, after genetic testing did not confirm an HAE diagnosis. The time to complete resolution of HAE symptoms for this patient’s attack was 497 h, which was included in the data analysis.

^d^ One patient (later determined to not have HAE) had 2 SAEs considered unrelated to treatment.

^e^ After treatment, 3 patients were determined to not have had a true HAE attack (pdC1-INH [*n* = 1]; PBO [*n* = 2]).

^f^ Patients in US or Canada only.

Two similarly designed randomized, double-blind, placebo (saline)-controlled trials evaluated rhC1-INH 50 U/kg (*n* = 12) or 100 U/kg (*n* = 29) as on-demand treatment for HAE attacks (Table 2) (41). Compared with placebo, rhC1-INH significantly reduced the time to onset of symptom relief (50 U/kg: median, 122 vs. 495 min, respectively; *P *= 0.01; 100 U/kg: median, 66 vs. 495 min, respectively; *P *< 0.001), as well as the time to minimal symptoms (50 U/kg: median, 247 vs. 1,210 min, respectively; *P *= 0.001; 100 U/kg: median, 266 vs. 1,210 min, respectively; *P *< 0.001). The median time to onset of symptom relief with IV C1-INH replacement therapies has varied in relation to trial design, product formulation, and dosing, with a range of 0.4–1.2 h for pdC1-INH and approximately 1.0–2.0 h for rhC1-INH (Table 2) (38, 39, 41). Review of these trials suggests that there is a dose response, with higher doses providing earlier onset of relief, as well as relief in a greater percentage of patients (42).

#### Short-term prophylaxis

Surgical and dental procedures and other medical interventions (e.g., diagnostic procedures) may trigger an attack in patients with HAE (43–46). WAO/EAACI guidelines recommend short-term prophylactic therapy for patients with HAE undergoing these types of procedures, with C1-INH replacement therapies considered the first-line option for short-term prophylaxis (13). Patient-specific emotional triggers may also precipitate an HAE attack, so WAO/EAACI guidelines also suggest that short-term prophylaxis be considered prior to exposure to an anticipated stressful life event.

No C1-INH formulation is currently approved in the United States for short-term prophylaxis, although pdC1-INH (Berinert and/or Cinryze) is approved for this indication in the European Union and Japan (Table 1). Evidence for the efficacy and safety of C1-INH replacement therapy for short-term prophylaxis is limited to retrospective and observational analyses, including from patient registries (Table 3) (40, 46–54). Data from a retrospective study (*N* = 137) reported that patients ≥2 years of age with HAE who received short-term prophylaxis with pdC1-INH (Berinert) prior to medical procedures experienced a decrease in post-procedure HAE attacks compared with the number of attacks they experienced before being diagnosed with HAE (Table 3) (46). It is noteworthy that patients in this study received pdC1-INH 500 U, which is half the dose noted by WAO/EACCI guidelines (1,000 U or 20 U/kg) for short-term, pre-procedure prophylaxis with pdC1-INH (13, 46). Registry data also indicated a low cumulative HAE attack rate within 3 days of pdC1-INH (Berinert) administration as short-term prophylaxis (47). Numerical trends suggested greater efficacy with weight-based doses of ≥15 IU/kg or absolute doses ≥1,500 IU. A recombinant preparation of C1-INH has also proved effective. A retrospective study (*N* = 51; 92% with type I HAE) demonstrated that short-term prophylaxis with rhC1-INH was efficacious for increasing the percentage of medical and dental procedures that remained attack-free compared with procedures in which no short-term prophylaxis was administered (Table 3) (48). However, rhC1-INH is currently not approved for prophylaxis (Table 1) (28).

**Table 3:** Summary of clinical trials of C1-INH replacement therapy as short-term or long-term prophylaxis for HAE attacks.

Therapy	Study and inclusion criteria	Treatments	Efficacy outcome(s)	Safety
pdC1-INH (Berinert)	**Short-term prophylaxis**			
	Farkas H, et al. (46) Retrospective analysis of pts with angioedema before HAE dx, and prospective analysis of the same pts after HAE dx receiving STP for medical procedures	STP with pdC1-INH 500 IU IV pre-procedure (*n* = 137) 20 pediatric pts (mean age, 12.2 y; range, 2.2–17.3 y) 117 adults (mean age, 41.3 y; range, 18.5–81.2 y)	HAE attacks decreased with pdC1-INH vs. pre-STP: 9% (5/54) vs. 58.7% (74/126)	No tx-related AEs No discontinuations
	Magerl M, et al. (47) Retrospective analysis of Berinert registry (US and Europe) 2010–2014	STP with pdC1-INH (median dose per infusion, 14.6 [range, 3.6–33.9] IU/kg or 1,000 [range, 500–3,500] IU) 79 patients (mean age, 42.4 y; range, 8–76 y)	Cumulative HAE attack rate after STP (attacks per infusion): Within 1 d: 0.04 (95% CI, 0.015–0.088) Within 2 d: 0.06 (95% CI, 0.028–0.115) Within 3 d: 0.11 (95% CI, 0.061–0.174)	6 AEs reported in 5/79 patients (6.3%), 2 (both headache) of which were considered tx related
	**Long-term prophylaxis**			
	Bork K and Hardt J (52) Prospective observational study End December 2008 Pts with type I or II HAE (*n* = 19) Mean age at end of study: 51.8 y (range, 35–78)	LTP with pdC1-INH IV ≥ once/wk Mean (SD) weekly dose: Tx onset: 1,253 U (641 U; range, 500–2,300 U) Tx end: 2,564 U (1,835 U; range, 500–7,000 U) Mean (SD) tx duration: 9 y (4.2); range, 4–19 y	8/14 pts had fewer attacks with LTP 12 mo after starting tx vs. pre-LTP 2 pts symptom-free 6 pts had 0.2–3.5 attacks/mo 5/14 pts had increase in attack frequency from beginning to end of study: Mean (SD) attacks/mo: beginning, 1.9 (0.8) vs. end, 9.7 (10.2) 1/14 pts discontinued after 5 y because of attacks	Not reported
	Craig T, et al. (53) MC, observational patient registry (US and Europe) 2010–2014 47 pts w/mean age 44.8 y (range, 13–79 y)	Total LTP duration for 47 pts: 430.2 mo Mean LTP duration: 9.2 mo/pt pdC1-INH IV dosing: Median absolute dose: 1,000 IU (range, 500–3,000 IU)	Pts with ≥1 attack within 7 d of tx: 68.1% (32/47) HAE attack rates among pts receiving pdC1-INH: 5.2 attacks/pt 0.06 attacks/infusion 0.6 attacks/mo Interval between pdC1-INH administration and attack: Mean (SD): 73.7 h (32.5 h) Median (range): 72.0 h (0–166.4 h)	Pts with ≥1 AE: 34.0% (16/47) Tx-related AEs: 3.7% (3/81) Not serious: Noncardiac chest pain (*n* = 1) Postinfusion headache (*n* = 1) SAE: Deep vein thrombosis (*n* = 1) SAEs not related to tx (*n* = 3): GI hemorrhage Severe HAE attack UTI No AE-related discontinuations
pdC1-INH (Cinryze)	**Long-term prophylaxis**			
	Zuraw BL, et al. (40) DB, PBO-C, crossover Pts ≥6 y with ≥2 HAE attacks/mo (moderate or severe attacks affecting abdomen, face, or external genitalia)	Two consecutive 12-wk tx periods pdC1-INH 1,000 U/10 ml (*n* = 11) or PBO (*n* = 11) administered IV q3–4 d	Mean number of HAE attacks during tx period (pdC1-INH vs. PBO): 6.3 vs. 12.7 (i.e., ∼2 vs. 4/mo) Mean difference in attack rates vs. PBO: 6.5 (95% CI, 4.2–8.7; *P *< 0.001) Mean (SD) duration of attacks (pdC1-INH vs. PBO): 2.1 (1.1) d vs. 3.4 (1.4); *P *= 0.002	AEs possibly related to pdC1-INH: Fever: 8.3% (1/12) Lightheadedness: 8.3% (1/12) Definitely related: Pruritus and rash: 8.3% (1/12)
	Zuraw BL and Kalfus I (49) OL, single arm Pts ≥1 y with ≥1 HAE attack/month, or any laryngeal attack Mean age: 36.5 y (range 3–82)	pdC1-INH 1,000 U administered IV q3–7 d at study site (*n* = 146) LTP duration up to 2.6 y	Number of HAE attacks/mo at baseline vs. during the study: Mean (SD): 4.7 (5.2) vs. 0.5 (0.8) 90% decrease from baseline Median (IQR): 3 (2–4) vs. 0.19 (0.0–0.64) 93.7% decrease from baseline	No AE-related study discontinuations 2 deaths not related to study drug (pulmonary arterial embolization of foreign material from IV injection of oral medication [*n* = 1]; HCC [*n* = 1]) 99/101 SAEs not related to pdC1-INH (2 of unknown relationship: musculoskeletal chest pain, major depression)
pdC1-INH (Haegarda)	**Long-term prophylaxis**			
	Longhurst H, et al. (50) COMPACT trial R, DB, PBO-C, crossover, ph 3 Pts ≥12 y with type I or II HAE w/ ≥4 attacks in 2-mo period ≤3 mo before screening Number of HAE attacks in 3 mo before screening, mean (SD): pdC1-INH 40 U/kg or 60 U/kg: 10.8 (6.7) or 8.8 (6.4) (i.e., ∼3 to 4/mo)	Two 16 wk tx periods: pdC1-INH 40 IU/kg bw SC (*n* = 45), or pdC1-INH 60 IU/kg bw SC (*n* = 45), followed by PBO, or vice versa, administered twice weekly	Number of HAE attacks per month: pdC1-INH 40 IU/kg (*n* = 43) vs. PBO (*n* = 44): 1.2 vs. 3.6 (*P *< 0.001; within-pt difference, −2.4) Mean/median decrease in attacks vs. PBO: 55%/89% pdC1-INH 60 IU/kg (*n* = 43) vs. PBO (*n* = 42): 0.5 vs. 4.0 (*P *< 0.001; within-pt difference, −3.5) Mean/median decrease in attacks vs. PBO: 84%/95% Pts with response (≥50% decrease in number of attacks with pdC1-INH 40 IU/kg or 60 IU/kg, vs. PBO): 76% or 90% Use of rescue medication/mo: pdC1-INH 40 IU/kg vs. PBO: 1.1 vs. 5.6 (*P *= 0.02; within-pt difference, −4.4) Mean/median decrease in use of rescue medication vs. PBO: 70%/89% pdC1-INH 60 IU/kg vs. PBO: 0.3 vs. 3.9 (*P *< 0.001; within-pt difference, −3.6) Mean/median decrease in use of rescue medication vs. PBO: 89%/100%	pdC1-INH 40 IU/kg (*n* = 43) pdC1-INH 60 IU/kg (*n* = 43) PBO (*n* = 86) AEs with onset ≤24 h of administration Any AE: pdC1-INH 40 IU, 67% (*n* = 29); pdC1-INH 60 IU/kg, 70% (*n* = 30), vs. PBO 66% (*n* = 57) Any tx-related AE: 33% (*n* = 14), 35% (*n* = 15), vs. 26% (*n* = 22) Any SAE: 2% (*n* = 1), 0, vs. 2% (*n* = 2) Any tx-related SAE: 0, 0, vs. 1% (*n* = 1) AEs leading to discontinuation: 0, 5% (*n* = 2), vs. 1% (*n* = 1) Nonserious AEs ≥5% of pts: Injection-site reaction: 28% (*n* = 12), 35% (*n* = 15), vs. 24% (*n* = 21) Dizziness: 9% (*n* = 4), 0, vs. 1% (*n* = 1) Upper respiratory tract infection: 7% (*n* = 3), 7% (*n* = 3), vs. 7% (*n* = 6) Hypersensitivity: 5% (*n* = 2), 7% (*n* = 3), vs. 1% (*n* = 1) Nasopharyngitis: 2% (*n* = 1), 19% (*n* = 8), vs. 7% (*n* = 6) Fatigue: 2% (*n* = 1), 2% (*n* = 1), vs. 7% (*n* = 6) Back pain: 2% (*n* = 1), 2% (*n* = 1), vs. 6% (*n* = 5)
	Craig T, et al. (51) OL extension of COMPACT trial R, OL, ph 3 December 2014–December 2017 Pts ≥6 y with type I or II HAE w/ ≥4 attacks in 2-mo period before enrollment into COMPACT trial or treatment-naïve pts Number of HAE attacks in 3 mo before screening, mean (SD): pdC1-INH 40 IU/kg: 12.8 (8.4) pdC1-INH 60 IU/kg: 12.7 (10.2) Overall, mean of 4.3 attacks/mo in the 3 mo before study entry	pdC1-INH 40 IU/kg bw SC (*n* = 63) pdC1-INH 60 IU/kg bw SC (*n* = 63)	745 HAE attacks (observation period of up to 2.7 y) Number of HAE attacks/mo (mean [SD]): pdC1-INH 40 IU/kg: 0.4 (0.7) pdC1-INH 60 IU/kg: 0.5 (0.9) Number of HAE attacks/y (median [range]): pdC1-INH 40 IU/kg: 1.3 (0.0–40.6) pdC1-INH 60 IU/kg: 1.0 (0.0–48.0) Time-normalized reduction in HAE attack rate from baseline (mean [SD]): pdC1-INH 40 IU/kg: −6.8 (−9.8 to −3.8) pdC1-INH 60 IU/kg: −6.8 (−10.9 to −2.7) Pts with response (≥50% decrease in number of attacks with pdC1-INH 40 IU/kg or 60 IU/kg) 94% or 92%	pdC1-INH 40 IU/kg (*n* = 63) pdC1-INH 60 IU/kg (*n* = 70)^a^ Any AE: pdC1-INH 40 IU/kg, 89% (*n* = 56); pdC1-INH 60 IU/kg, 83% (*n* = 58) Events (events/patient-year): pdC1-INH 40 IU/kg, 948 (11.3); pdC1-INH 60 IU/kg, 849 (8.5) Any SAE: pdC1-INH 40 IU/kg, 6% (*n* = 4); pdC1-INH 60 IU/kg, 7% (*n* = 5) Any tx-related SAE: 0 (both groups) AEs leading to discontinuation: pdC1-INH 40 IU/kg, 0; pdC1-INH 60 IU/kg, 1% (*n* = 1) Most common AEs (pdC1-INH 40 IU/kg, pdC1-INH 60 IU/kg) Nasopharyngitis: 19% (*n* = 12), 30% (*n* = 21) Injection-site pain: 27% (*n* = 17), 14% (*n* = 10) Injection-site erythema: 16% (*n* = 10), 17% (*n* = 12) Headache: 16% (*n* = 10), 14% (*n* = 10) Injection-site bruising: 14% (*n* = 9), 10% (*n* = 7) UTI: 13% (*n* = 8), 11% (*n* = 8)
rhC1-INH (Ruconest)	**Short-term prophylaxis**			
	Valerieva A, et al. (48) Retrospective study Pts with HAE (51 pts received rhC1-INH) Median age 44 y (range, 17–73) Procedures (e.g., medical/dental) with rhC1-INH prophylaxis (*n* = 70) vs. procedures with no prophylaxis (*n* = 26)	Median rhC1-INH IV dose: 3,075 IU (range, 2,100–4,200 IU) Administered median 60 min pre-procedure	rhC1-INH vs. no rhC1-INH Attack-free procedures 2 d post-procedure: 97.1% vs. 23.1% 7 d post-procedure: 88.6% vs. 19.2%	No AEs in the 70 treated procedures
	**Long-term prophylaxis**			
	Riedl MA, et al. (54) R, DB, PBO-C, crossover, ph 2 Pts ≥13 y with HAE w/ ≥4 attacks/mo for 3 consecutive mo before study initiation (*n* = 32) Mean (SD) attacks ≤3 mo: 17.9 (7.2) Median (range) attacks ≤3 mo: 14.5 (12–33)	Three 4-wk tx periods, with 1-wk washout before crossover: rhC1-INH 50 IU/kg bw (pts <84 kg) or 4,200 IU (pts ≥84 kg) once or twice weekly IV, vs. PBO	Mean (SD) number of HAE attacks in each 4-wk tx period (*n* = 31): Once weekly, 4.4 (3.2) Twice weekly: 2.7 (2.4) PBO: 7.2 (3.6) Mean differences vs. PBO: −2.8 (*P *= 0.0004) and −4.4 (*P *< 0.0001), respectively Mean decrease in attack frequency (once weekly, twice weekly vs. PBO) (*n* = 31): 34.9% and 63.3% Pts with clinical response (≥50% decrease in number of attacks with rhC1-INH vs. PBO): Once weekly tx: 13/31 (42%) Twice weekly tx: 23/31 (74%)	rhC1-INH once weekly (*n* = 29) rhC1-INH twice weekly (*n* = 29) PBO (*n* = 28) Any AEs: once weekly, 45% (*n* = 13); twice weekly, 34% (*n* = 10) vs. PBO 29% (*n* = 8) Tx-related AEs: 0, 7% (*n* = 2), vs. 0 SAEs: 0, 3% (*n* = 1), vs. 0 AEs occurring in ≥5% pts: Nasopharyngitis: 10% (*n* = 3), 0, vs. 7% (*n* = 2) Headache: 7% (*n* = 2), 17% (*n* = 5), vs. 0 Anxiety: 7% (*n* = 2), 0, vs. 0

AE, adverse event; bw, body weight; C1-INH, C1 esterase inhibitor; COMPACT, Optimal Management of Preventing Angioedema with Low-Volume Subcutaneous C1-Inhibitor Replacement Therapy; DB, double-blind; dx, diagnosis; GI, gastrointestinal; HAE, hereditary angioedema; HAE-nC1, normal hereditary angioedema; HCC, hepatocellular carcinoma; IQR, interquartile range; IV, intravenous; LTP, long-term prophylaxis; MC, multicenter; OL, open-label; PBO, placebo; PBO-C, placebo-controlled; pdC1-INH, plasma-derived C1 esterase inhibitor; ph, phase; pts, patients; R, randomized; rhC1-INH, recombinant human C1 esterase inhibitor; SAE, serious adverse event; SC, subcutaneous; STP, short-term prophylaxis; tx, treatment; UTI, urinary tract infection.

^a^ Included 7 patients whose treatment was uptitrated from 40 IU/kg to 60 IU/kg and were included in both treatment arms in the safety population.

#### Long-term prophylaxis

WAO/EAACI guidelines recommend consideration of long-term prophylaxis for all patients with type I or II HAE, taking into consideration patients’ preferences, disease activity, HAE impact on quality of life, availability of health care resources, and inability to achieve adequate control of symptoms with appropriate on-demand therapy (13). Patients with HAE should be evaluated for long-term prophylaxis at each office or telemedicine visit or at least annually because these factors can vary over time. C1-INH replacement (e.g., pdC1-INH) is recommended as a first-line, long-term prophylactic therapy (13). C1-INH products approved in the United States and the European Union for routine, long-term prophylaxis include IV pdC1-INH (Cinryze) and a subcutaneous (SC) formulation of pdC1-INH (Haegarda; CSL Behring LLC; Table 1).

A double-blind, placebo-controlled, crossover trial (*N* = 22) determined that pdC1-INH (Cinryze) 1,000 U IV administered every 3 to 4 days as long-term prophylaxis significantly reduced the HAE attack rate compared with placebo during a 12-week period (difference, 6.5 attacks; *P *< 0.001; Table 3) (40). In addition, HAE attack duration was significantly decreased for patients receiving pdC1-INH prophylaxis compared with those receiving placebo (2.1 vs. 3.4 days, respectively; *P *= 0.002; Table 3). An open-label prophylaxis trial (*N* = 146) of pdC1-INH 1,000 U (Cinryze) every 3–7 days for up to 2.6 years found a 90% decrease from baseline in the mean number of attacks [4.7 attacks/month (baseline) vs. 0.5 attacks/month] (49). During that trial, 140 patients underwent complement testing to assess C1-INH antigen levels and functional activity at baseline (immediately prior to treatment) and 1 h after treatment. Baseline functional activity was inversely correlated with HAE attack frequency (i.e., lower baseline C1-INH activity was predictive of an increased attack rate during treatment; *R *= 0.2; *P *= 0.01); however, no significant relationship was observed between postinjection C1-INH function and attack frequency (*R *= 0.09; *P *= 0.3) (49).

In a phase 3, randomized, double-blind, crossover trial (*N* = 90), a significant decrease in the mean frequency of attacks was observed with SC pdC1-INH 40 or 60 IU/kg, administered twice weekly for 16 weeks, compared with placebo (Table 3; *P *< 0.001 for both doses vs. placebo) (50). In that trial, 76% and 90% of patients were responders (i.e., ≥50% decrease in the number of attacks vs. placebo) with pdC1-INH 40 and 60 IU/kg, respectively. An open-label extension trial (*N* = 126) supported these results, with >90% of patients in the pdC1-INH 40- and 60-IU/kg treatment groups achieving a ≥50% reduction from baseline in HAE attacks (Table 3) (51). Pharmacokinetic modeling indicated that pdC1-INH administered SC exhibits a more consistent exposure and a lower peak-to-trough ratio compared with pdC1-INH given IV, which may account for the improved efficacy of SC pdC1-INH observed in clinical trials (50, 55).

A prospective, observational trial of long-term HAE prophylaxis with IV pdC1-INH (Berinert), which is not currently approved for this indication, reported that more than half of the patients (8/14) experienced a reduction in attack frequency during the last 12 months of treatment (mean duration, 9 years) compared with the attack frequency before initiation of long-term prophylactic therapy (Table 3) (52). However, 5 of the 14 patients experienced an increase in attack frequency, even with increased pdC1-INH dosing, and 1 patient discontinued participation in the trial after 5 years because of attack frequency (after receiving pdC1-INH 1,000 U daily for 1.5 years). One hypothesis for the increase in HAE attack rate during the trial was that frequent IV injections may be activating the plasma contact system, either directly or indirectly, leading to an increase in underlying disease activity that is otherwise masked by the effectiveness of pdC1-INH administration (52). Patients in this trial received pdC1-INH at least once weekly for a mean of 9 years (range, 4‒19 years). Cycling from increased C1-INH levels immediately after treatment to a decrease in C1-INH levels several days post-treatment may have lowered the threshold for activation of the plasma contact system (52). An observational registry trial (*N* = 47) of long-term HAE prophylaxis with pdC1-INH (Berinert; mean 9.2 months of treatment per patient) showed that more than two-thirds of patients (68.1%) experienced ≥1 attack within 7 days of receiving IV treatment (Table 3) (53).

rhC1-INH is also not approved as long-term prophylaxis of HAE (Table 1) but proved efficacious for this use in a phase 2, randomized, double-blind, placebo-controlled crossover trial (Table 3) (54). When administered once or twice weekly for 4 weeks, rhC1-INH reduced in a statistically significant manner the mean number of attacks, as well as the attack frequency, compared with placebo (54).

## Single pathway therapies

**Figure 2:**
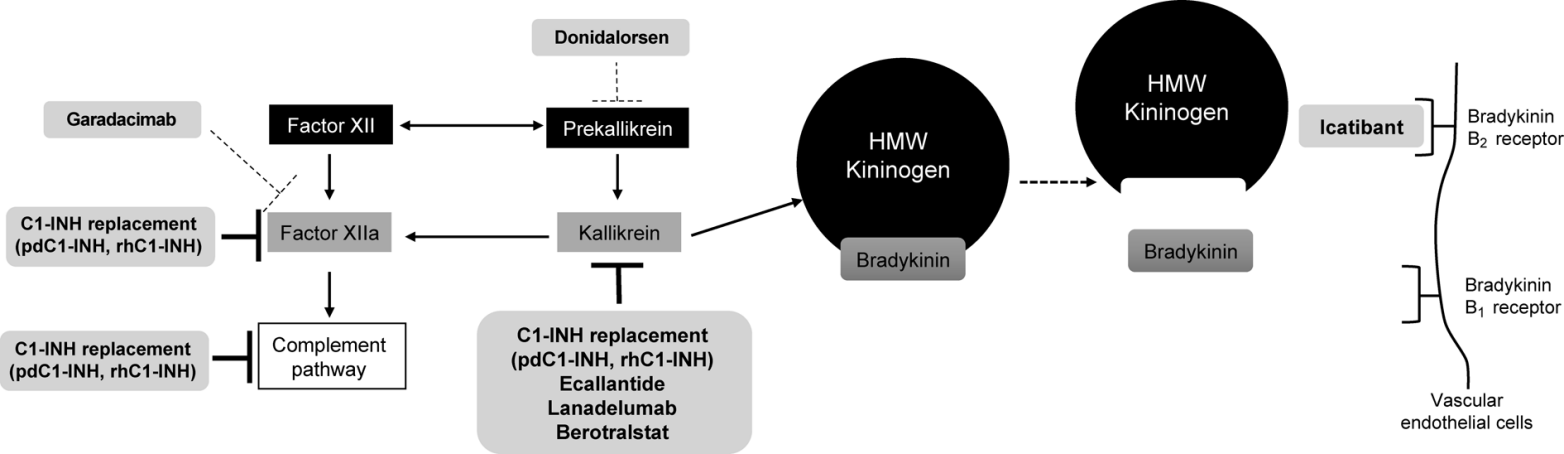
Therapeutic targeting of pathways in HAE. Inhibitors of the pathway are shown in light gray shaded boxes at their respective sites of action. Investigational therapies are shown with dashed lines at their target sites. *C1-INH, C1 esterase inhibitor; HMW, high molecular weight; pdC1-INH, plasma-derived C1-INH; rhC1-INH, recombinant human C1-INH*. *Figure created with data from Cicardi M, et al. J Allergy Clin Immunol Pract. (2018) 6(4):1132–41; Zuraw BL. N Engl J Med. (2008) 359(10):1027–36; and Fijen LM, et al. Clin Rev Allergy Immunol. (2021) 61(1):66–7* (5, 14, 16).

Single pathway therapies that are currently available for the treatment of HAE include the SC administered B2 receptor antagonist icatibant (Firazyr; Takeda Pharmaceuticals America, Inc.; Lexington, MA), the SC administered plasma kallikrein inhibitors ecallantide (Kalbitor; Dyax Corp., a Takeda company; Lexington, MA) for attacks or lanadelumab (Takhzyro; Dyax Corp; Lexington, MA) for prophylaxis, as well as the oral plasma kallikrein inhibitor berotralstat (Orladeyo; BioCryst Pharmaceuticals, Inc.; Durham, NC; Table 1), also for prophylaxis. By blocking the effects of bradykinin at the B2 receptor (Figure 2), icatibant has been shown to reverse the vascular permeability observed in C1-INH gene knockout mice (56) and to decrease bradykinin-induced vasodilation in the forearms of healthy volunteers (57). Treatment with icatibant also decreased bradykinin levels in patients with HAE, but changes in plasma bradykinin levels were not directly related to the degree of symptom relief (58). Working upstream from icatibant, plasma kallikrein inhibitors block the binding site of kallikrein to prevent cleavage of HMW kininogen and subsequent bradykinin release and also reduce further activation of factor XIIa to disrupt the positive feedback loop that would otherwise lead to increased kallikrein production (Figure 2) (16, 33, 59, 60).

### On-demand therapy for HAE attacks

In addition to IV C1-INH replacement therapy, WAO/EAACI guidelines recommend IV ecallantide or icatibant as on-demand treatment of HAE attacks (13). Both agents are approved in the United States for this indication (Table 1), while icatibant is approved in the European Union (61) and Japan (62).

In two phase 3 randomized, double-blind trials (For Angioedema Subcutaneous Treatment [FAST]-1 [*N* = 56] and FAST-2 [*N* = 74]), icatibant 30 mg SC was administered for the treatment of acute HAE attacks. Icatibant reduced the median time to clinically significant relief of the patient’s index symptom compared with oral tranexamic acid 3 g daily for 2 days (FAST-2; 2.0 vs. 12.0 h, respectively; *P *< 0.001) and also reduced the median time compared with placebo, although the difference was not statistically significant (FAST-1; 2.5 vs. 4.6 h; *P *= 0.1; Table 4) (63–70). Furthermore, in both trials, icatibant significantly reduced the median time to initial symptom improvement by both patient assessment and investigator assessment. In a third phase 3 trial, icatibant 30 mg SC (*n* = 43) was significantly more efficacious than placebo (*n* = 45) for reducing the median time to onset of symptom relief for nonlaryngeal attacks (2.0 h vs. 19.8 h, respectively; *P *< 0.001) (65).

**Table 4:** Summary of clinical trials of bradykinin B_2_ receptor antagonists and kallikrein inhibitors as on-demand or prophylactic treatment of HAE attacks.

Therapy	Study and inclusion criteria	Treatments	Efficacy outcome(s)	Safety
**On-Demand Treatment**				
**Bradykinin B_2_ Receptor Antagonist**				
Icatibant (Firazyr)	Cicardi M, et al. (64) FAST-1 and FAST-2 DB, R, C, ph 3 Pts ≥18 y with type I or II HAE (abdominal or cutaneous attacks)	FAST-1: Icatibant 30 mg SC (*n* = 27) PBO (*n* = 29) FAST-2: Icatibant 30 mg SC (*n* = 36) Oral tranexamic acid 3 g/d for 2 d (*n* = 38)	FAST-1: icatibant vs. PBO FAST-2: icatibant vs. tranexamic acid Median (IQR) time to clinically significant relief of index symptom: FAST-1: 2.5 h (1.1–6.0) vs. 4.6 h (1.8–10.2; *P *= 0.1) FAST-2: 2.0 h (1.0–3.5) vs. 12.0 h (3.5–25.4; *P *< 0.001) Pts (95% CI) with clinically significant relief of index symptom 4 h after start of tx: FAST-1: 67% (46–84) vs. 46% (28–66; *P *= 0.2) FAST-2: 80% (63–92) vs. 31% (16–48; *P *< 0.001) Median (IQR) time to first symptom improvement (pt assessed) FAST-1: 0.8 h (0.5–2.0) vs. 16.9 h (3.2–NA; *P *< 0.001) FAST-2: 0.8 h (0.4–1.4) vs. 7.9 h (1.1–NA; *P *< 0.001) Median (IQR) time to first symptom improvement (investigator assessed) FAST-1: 1.0 h (0.8–2.0) vs. 5.7 h (2.0–11.2; *P *< 0.001) FAST-2: 1.5 h (0.7–3.0) vs. 6.9 h (4.0–13.8; *P *< 0.001)	FAST-1: icatibant (*n* = 27) vs. PBO (*n* = 29) FAST-2: icatibant (*n* = 36) vs. tranexamic acid (*n* = 38) Any AEs: FAST-1: 44% (*n* = 12) vs. 66% (*n* = 19) FAST-2: 53% (*n* = 19) vs. 42% (*n* = 16) Tx-related AEs: FAST-1: 15% (*n* = 4) vs. 3% (*n* = 1) FAST-2: 14% (*n* = 5) vs. 11% (*n* = 4) SAEs: FAST-1: 0 vs. 0 FAST-2: 11% (*n* = 4) vs. 3% (*n* = 1) Injection-site reaction: FAST-1: 96% (*n* = 26) vs. 28% (*n* = 8) FAST-2: 97% (*n* = 35) vs. 26% (*n* = 10)
	Lumry WR, et al. (65) FAST-3 MC, R, DB, PBO-C, ph 3 Pts ≥18 y with type I or II HAE (abdominal, cutaneous, or laryngeal attacks)	Icatibant 30 mg SC (*n* = 46) vs. PBO (*n* = 47) 3/46 icatibant and 2/47 PBO pts had laryngeal attacks Tx administered ≤6 h of attack	Median (95% CI) time to onset of symptom relief (icatibant vs. PBO): Nonlaryngeal attacks^a^: 2.0 h (1.5–3.0) vs. 19.8 h (6.1–26.3; *P *< 0.001) Laryngeal attacks^b^: 2.5 h (1.3–3.0) vs. 3.2 h (1.0–5.4) Median (95% CI) time to first symptom improvement (pt-assessed) Nonlaryngeal attacks: 0.8 h (0.5–1.0) vs. 3.5 h (1.9–5.4; *P *< 0.001) Laryngeal attacks: 1.0 h (0.6–2.5) vs. 2.1 h (0.5–3.9) Median (95% CI) time to first symptom improvement investigator-assessed) Nonlaryngeal attacks: 0.8 h (0.6–1.3) vs. 3.4 h (2.6–6.0; *P *< 0.001) Laryngeal attacks: 1.0 h (0.6–2.5) vs. 2.2 h (0.5–3.9)	Icatibant 30 mg SC (*n* = 46) PBO (*n* = 46) ≥1 AE: 41.3% (*n* = 19) vs. 52.2% (*n* = 24) ≥1 drug-related AE: 10.9% (*n* = 5) vs. 6.5% (*n* = 3) ≥1 SAE: 0 vs. 6.5% (*n* = 3) ≥1 tx-related SAE: 0 vs. 0 AE-related deaths: 0 vs. 2.2%^c^ (*n* = 1)
**Kallikrein Inhibitor**				
Ecallantide (Kalbitor)	Cicardi M, et al. (66) EDEMA3 trial 8 December 2005–10 February 2007 MC, R, DB, PBO-C, ph3 Pts ≥10 y with HAE (GI, laryngeal, peripheral attacks)	Ecallantide 30 mg SC (*n* = 36) PBO (*n* = 36) Pts observed for ≥4 h after tx	TOS^d^ at 4 h post-tx (ecallantide vs. PBO): Mean (SD): 46.8 (59.3) vs. 21.3 (69.0) Median (range): 50.0 (−100.0 to 100.0) vs. 0 (−100.0 to 100.0; *P *= 0.004) Pts with significant improvement in overall response ≤4 h: 50% (*n* = 18) vs. 33% (*n* = 12)	Ecallantide 30 mg SC (*n* = 36) PBO (*n* = 36) ≥1 AE: 56% (*n* = 20) vs. 33% (*n* = 12) ≥1 tx-related AE: 11% (*n* = 4) vs. 14% (*n* = 5) SAEs: 8% (*n* = 3) vs. 6% (*n* = 2) Most common AEs: Headache: 11% (*n* = 4) vs. 6% (*n* = 2) Diarrhea: 8% (*n* = 3) vs. 0 Pyrexia: 8% (*n* = 3) vs. 0 Nasopharyngitis: 6% (*n* = 2) vs. 3% (*n* = 1) Tachycardia, not otherwise specified: 6% (*n* = 2) vs. 3% (*n* = 1) Nasal congestion: 6% (*n* = 2) vs. 0 No AE-related mortality or study discontinuations
	Levy RJ, et al. (67) EDEMA4 trial DB, R, PBO-C, ph 3 Pts ≥10 y with type I or II HAE (any attack location)	Ecallantide 30 mg SC (*n* = 48) PBO (*n* = 48) Pts observed for ≥4 h after tx	TOS^d^ at 4 h post-tx (ecallantide vs. PBO): Mean (SD): 53.4 (49.7) vs. 8.1 (63.2) Median (range): 50.0 (−66.7 to 100) vs. 0 (−100.0 to 100.0; *P *= 0.003) Pts maintaining significant improvement in overall response through 24 h: 44% (*n* = 21) vs. 21% (*n* = 10; *P *= 0.02)	Ecallantide 30 mg SC (*n* = 48) PBO (*n* = 48) ≥1 AE: 17% (*n* = 8) vs. 40% (*n* = 19) Most common AEs: Nausea: 6% (*n* = 3) vs. 2% (*n* = 1) Headache: 4% (*n* = 2) vs. 10% (*n* = 5) Dizziness: 4% (*n* = 2) vs. 2% (*n* = 1) Abdominal pain: 2% (*n* = 1) vs. 4% (*n* = 2)
**Prophylactic Treatments**				
**Kallikrein Inhibitors**				
Lanadelumab (Takhzyro)	Banerji A, et al. (68) HELP trial MC, R, DB, *P*, PBO-C, ph 3 Pts ≥12 y with type I or II HAE with ≥1 attack/q4 wk	Lanadelumab (26 wk tx) 150 mg SC q4 wk (*n* = 28) 300 mg SC q4 wk (*n* = 29) 300 mg SC q2 wk (*n* = 27) PBO (*n* = 41) Run-in period attack rate (mean attacks/mo [SD]): Lanadelumab 150 mg q4 wk: 3.2 (1.8) 300 mg q4 wk: 3.7 (2.5) 300 mg q2 wk: 3.5 (2.3) PBO: 4.0 (3.3)	Mean (95% CI) number of attacks/mo Lanadelumab 150 mg q4 wk: 0.5 (0.3–0.7) 300 mg q4 wk: 0.5 (0.4–0.8) 300 mg q2 wk: 0.3 (0.1–0.5) PBO: 2.0 (1.6–2.4) Mean difference (95% CI) in number of attacks/mo vs. PBO 150 mg q4 wk: −1.5 (−1.9 to −1.1; *P *< 0.001) 300 mg q4 wk: −1.4 (−1.8 to −1.0; *P *< 0.001) 300 mg q2 wk: −1.7 (−2.1 to −1.3; *P *< 0.001)	Lanadelumab 150 mg q4 wk (*n* = 28) 300 mg q4 wk (*n* = 29) 300 mg q2 wk (*n* = 27) PBO (*n* = 41) Any AE: 150 mg q4 wk, 89.3% (*n* = 25); 300 mg q4 wk, 86.2% (*n* = 25); 300 mg q2 wk, 96.3% (*n* = 26), vs. PBO 75.6% (*n* = 31) Any tx-related AE: 60.7% (*n* = 17), 48.3% (*n* = 14), 70.4% (*n* = 19), vs. 34.1% (*n* = 14) Injection site pain: 42.9% (*n* = 12), 31.0% (*n* = 9), 51.9% (*n* = 14), vs. 26.8% (*n* = 11) Any SAE: 0, 10.3% (*n* = 3), 3.7% (*n* = 1), vs. 0 Any AE leading to discontinuation: 0, 3.4% (*n* = 1), 0, vs. 2.4% (*n* = 1)
	Banerji A, et al. (69) OL extension trial Pts ≥12 y with type I or II HAE with ≥1 attack/q4 wk Planned treatment duration: 924 d	Rollovers from HELP trial (*n* = 109): Lanadelumab 300 mg (Day 0; last visit HELP trial); treatment paused until first HAE attack (dose-and-wait stage), at which point patients received second dose lanadelumab 300 mg and continued lanadelumab 300 mg q2 wk (regular dosing stage) Non-rollovers (*n* = 103): lanadelumab 300 mg q2 wk Mean (SD) lanadelumab exposure: 29.6 (8.2) months; 92.5% of patients completed ≥12 mo	Reduction in attack rate Overall: 87.4% Rollovers (starting from regular dosing stage/second dose, not including first HAE attack): 92.4% Non-rollovers (starting from Day 0): 82.0% Attack-free (overall): Mean (SD), % days: 97.7% (6.0%) Mean, (SD), duration: 14.8 mo (415.0 d) ≥6 mo: 81.8% ≥12 mo: 68.9%	Total (*N* = 212) Any AE: 97.2% (*n* = 206) Treatment-related AE: 54.7% (*n* = 116) Any SAE: 9.9% (*n* = 21) Any treatment-related SAE: 0 Any severe AE: 17.9% (*n* = 38) Any investigator-reported AESIs, including hypersensitivity reactions and disordered coagulation (hypercoagulability events and bleeding events): 6.1% (*n* = 13) Any hospitalizations due to AEs: 9.9% (*n* = 21) Any AE leading to discontinuation: 2.8% (*n* = 6) Most common AEs: Injection site pain: 47.2% (*n* = 100) Viral URTI: 42.0% (*n* = 89) Upper respiratory tract infection: 25.9% (*n* = 55) Headache: 24.5% (*n* = 52) Injection site erythema: 17.0% (*n* = 36) Arthralgia: 12.7% (*n* = 27)
Berotralstat (Orladeyo)	Zuraw B, et al. (63) APeX-2 trial, part 1 14 March 2018–10 April 2019 (first 24 weeks of treatment) MC, R, DB, *P*, PBO-C, ph 3 Pts aged ≥12 y (US and Canada) or ≥18 y (Europe) with type I or II HAE with ≥2 HAE attacks requiring treatment or causing functional impairment in first 56 d of run-in period	Berotralstat orally once daily for 24 wk 110 mg (*n* = 41) 150 mg (*n* = 40) PBO (*n* = 40, 39 dosed) Mean (SD) baseline HAE attacks/28-d period over screening period of up to 70 d (investigator confirmed) Berotralstat 110 mg: 2.97 (1.36) 150 mg: 3.06 (1.56) PBO: 2.91 (1.12)	Mean number of investigator-confirmed attacks/mo over 24 wk Berotralstat 110 mg: 1.65 150 mg: 1.31 PBO: 2.35 Attack rate ratio (95% CI) relative to PBO Berotralstat 110 mg: 0.70 (0.51–0.95), *P *= 0.024 150 mg: 0.56 (0.41–0.77), *P *< 0.001	Berotralstat 110 mg (*n* = 41) Berotralstat 150 mg (*n* = 40) PBO (*n* = 39) Any AE: 83% (*n* = 34)/85% (*n* = 34)/77% (*n* = 30) Any SAE: 2% (*n* = 1)/0/8% (*n* = 3) Tx-related AEs: 0 (all groups) AE leading to discontinuation: 7% (*n* = 3)/3% (*n* = 1)/3% (*n* = 1) Most common AEs (berotralstat 110 mg/berotralstat 150 mg/PBO): URTI: 32% (*n* = 13)/30% (*n* = 12)/28% (*n* = 11) Nausea: 15% (*n* = 6)/15% (*n* = 6)/18% (*n* = 7) Abdominal pain: 10% (*n* = 4)/23% (*n* = 9)/10% (*n* = 4) Vomiting: 10% (*n* = 4)/15% (*n* = 6)/3% (*n* = 1) Diarrhea: 10% (*n* = 4)/15% (*n* = 6)/0 Headache: 7% (*n* = 3)/10% (*n* = 4)/5% (*n* = 2) Back pain: 2% (*n* = 1)/10% (*n* = 4)/3% (*n* = 1)
	Wedner HJ, et al. (70) APeX-2 trial, part 2 14 March 2018–25 September 2019 (first 48 wk of treatment) MC, R, DB, *P*, ph3 Pts aged ≥12 y (US and Canada) or ≥18 y (Europe) with type I or II HAE with ≥2 HAE attacks requiring treatment or causing functional impairment in first 56 d of run-in period	Pts taking berotralstat in part 1 continued same dose; pts taking PBO re-randomized to berotralstat 110 or 150 mg once daily (all blinded) Berotralstat once daily for 48 wk 110 mg (*n* = 37) 150 mg (*n* = 37) PBO for 24 weeks, then berotralstat for 24 wk 110 mg (*n* = 17) 150 mg (*n* = 17) Mean (SE) baseline attack rate (attacks/mo): Berotralstat 110 mg: 2.97 (0.21) Berotralstat 150 mg: 3.06 (0.25) Mean (SE) attack rate at week 24 (before berotralstat) PBO → berotralstat 110 mg: 2.39 (0.41) PBO → berotralstat 150 mg: 2.56 (0.61)	Mean (SEM) number of investigator-confirmed attacks/month at 48 wk: Berotralstat 110 mg: 1.35 (0.33) Berotralstat 150 mg: 1.06 (0.25) PBO → berotralstat 110 mg: 1.25 (0.32) PBO → berotralstat 150 mg: 0.57 (0.23)	Berotralstat 110 mg (*n* = 41) Berotralstat 150 mg (*n* = 40) Results up to wk 48: Any AE: 92.7% (*n* = 38)/95.0% (*n* = 38) Any SAE: 2.4% (*n* = 1)/2.5% (*n* = 1) Tx-related SAE: 0 (both groups) AE-related discontinuation: 9.8% (*n* = 4)/7.5% (*n* = 3) Investigator-identified rash 0/5.0% (*n* = 2) Most common AEs (berotralstat 110 mg/berotralstat 150 mg): URTI: 36.6% (*n* = 15)/52.5% (*n* = 21) Nausea: 19.5% (*n* = 8)/20.0% (*n* = 8) Abdominal pain: 9.8% (*n* = 4)/30.0% (*n* = 12) Dyspepsia: 9.8% (*n* = 4)/12.5% (*n* = 5) Diarrhea: 12.2% (*n* = 5)/17.5% (*n* = 7) Vomiting: 9.8% (*n* = 4)/15.0% (*n* = 6) Headache: 9.8% (*n* = 4)/12.5% (*n* = 5) Flatulence: 7.3% (*n* = 3)/7.5% (*n* = 3) Back pain: 4.9% (*n* = 2)/12.5% (*n* = 5) Gastroesophageal reflux disease: 9.8% (*n* = 4)/5.0% (*n* = 2)

AE, adverse event; AESI, adverse event of special interest; APeX-2, Angioedema Prophylaxis 2; C, controlled; DB, double-blind; EDEMA, Evaluation of DX88’s Effects in Mitigating Angioedema; FAST, For Angioedema Subcutaneous Treatment; GI, gastrointestinal; HAE, hereditary angioedema; HELP, Hereditary Angioedema Long-term Prophylaxis; IQR, interquartile range; MC, multicenter; NA, not available; *P*, parallel group; PBO, placebo; PBO-C, placebo-controlled; ph, phase; pts, patients; R, randomized; SAE, serious adverse event; SC, subcutaneous; TOS, treatment outcome score; tx, treatment; URTI, upper respiratory tract infection; VAS, visual analog scale.

^a^ Symptom relief defined as 50% decrease from pretreatment in VAS-3 score; VAS-3 is a 3-symptom composite visual analog scale score for cutaneous and/or abdominal attacks that includes scores for skin swelling, skin pain, and abdominal pain.

^b^ Symptom relief defined as 50% decrease from pretreatment in VAS-5 score; VAS-5 is a 5-symptom composite visual analog scale score for laryngeal attacks that includes scores for skin swelling, skin pain, abdominal pain, difficulty swallowing, and voice change.

^c^ Myocardial infarction.

^d^ Score is a composite of patient-reported outcomes related to site(s) of symptoms, baseline symptom severity, and treatment response. Scores range from +100 (significant improvement in symptoms) to -100 (significant worsening of symptoms).

In a phase 3, randomized, double-blind trial (*N* = 72), on-demand treatment with ecallantide 30 mg SC significantly improved the median treatment outcome score (patient-reported composite comprising sites of symptoms, symptom severity, and response to treatment) compared with placebo (Table 4; *P *= 0.004) (66). Further, a greater percentage of patients who received ecallantide experienced significant improvement (“a lot better or resolved”) in overall response vs. placebo (50% vs. 33%, respectively) (66). Results of another phase 3, randomized, double-blind trial (*N* = 96) noted a significant improvement in treatment outcome score 4 h after dosing with ecallantide compared with placebo (*P *= 0.003) and an overall response maintained through 24 h (*P *= 0.02; Table 4) (67). Although anaphylaxis was not reported during these 2 trials (66, 67), a retrospective database analysis of ecallantide clinical trials identified 8 of 230 patients (3.5%) with hypersensitivity reactions consistent with anaphylaxis (71). All of these events occurred within 1 h of ecallantide administration, and no cases of anaphylaxis occurred after the first exposure (71). Accordingly, the US ecallantide prescribing information contains a boxed warning regarding the potential for anaphylaxis and states that only health care providers should administer the medication (33).

### Long-term prophylaxis

The plasma kallikrein inhibitors lanadelumab and berotralstat are both recommended by WAO/EAACI guidelines as first-line options for long-term prophylaxis (13) and are approved in the United States, European Union, and Japan for this indication (Table 1). A phase 3 trial (*N* = 125) demonstrated that the 3 different lanadelumab SC dosing regimens examined (150 mg every 4 weeks, 300 mg every 4 weeks, or 300 mg every 2 weeks) all significantly reduced the mean number of attacks experienced by patients each month compared with placebo (*P *< 0.001 for all comparisons) over the 26-week treatment period (Table 4) (68). Patients who completed the trial (*n* = 109) or new patients (*n* = 103) were eligible for an open-label extension trial of lanadelumab 300 mg SC every 2 weeks (Table 4) (69). Long-term treatment with lanadelumab reduced the mean HAE attack rate by 87.4% overall. During treatment, patients were attack-free for 97.7% of days on average, and the mean duration of the attack-free period was >14 months. More than 80% of patients remained attack-free for ≥6 months and 69% were attack-free for ≥12 months.

In part 1 (a 24-week, placebo-controlled phase) of a phase 3, randomized, double-blind trial (*N* = 121), berotralstat 110 and 150 mg once daily significantly reduced the mean number of HAE attacks per month compared with placebo (*P *= 0.024 and *P *< 0.001, respectively; Table 4) (63). The attack rate ratio relative to placebo (95% CI) was 0.70 (0.51–0.95) for berotralstat 110 mg and 0.56 (0.41–0.77) for berotralstat 150 mg. Both doses of berotralstat significantly reduced the rate of HAE attacks in patients with ≥2 attacks per month at baseline (110 mg, *P *= 0.04; 150 mg, *P *= 0.005), but only berotralstat 150 mg significantly reduced the HAE attack rate among patients with <2 attacks per month at baseline (*P *= 0.009). In part 2 of this trial, berotralstat-treated patients in part 1 continued their assigned double-blind dose, and placebo-treated patients were re-randomized to double-blind berotralstat 110 mg or 150 mg once daily (Table 4) (70). Among patients who continued on berotralstat, the mean (SE) monthly attack rates declined from 2.97 (0.21) at baseline to 1.35 (0.33) at week 48 in the berotralstat 110-mg group and from 3.06 (0.25) at baseline to 1.06 (0.25) at week 48 in the berotralstat 150-mg group. For placebo-treated patients who switched to berotralstat, the decrease in HAE attack rates was similar to that observed in part 1 of the trial in the berotralstat treatment groups (Table 4) (70).

## Investigational treatments

Garadacimab (CSL312) is a human recombinant monoclonal antibody that inhibits factor XIIa and thus acts on the complement pathway in addition to the kallikrein-kinin pathway (Figure 2) (72). It has been shown to potently inhibit bradykinin formation in plasma samples from patients with HAE and to reduce edema in animal models (72). Data have been published from a phase 2, randomized, double-blind trial (73). Patients were randomly assigned to receive an IV loading dose (placebo or garadacimab 40, 100, or 300 mg) followed by SC administration (placebo [*n* = 8], garadacimab 75 mg [*n* = 9], garadacimab 200 mg [*n* = 8], or garadacimab 600 mg [*n* = 7]) on day 6 and then every 4 weeks for 12 weeks. During the 12-week SC treatment period, the median number (interquartile range) of monthly attacks was 4.6 (3.1–5.0) in the placebo group, 0.0 (0.0–0.4) in the garadacimab 75-mg group, 0.0 (0.0–0.0) in the garadacimab 200-mg group, and 0.3 (0.0–0.7) in the garadacimab 600-mg group. Compared with placebo, garadacimab 75 mg, 200 mg, and 600 mg significantly reduced the median attack rate by 100% (*P *= 0.0002, *post hoc* analysis), 100% (*P *= 0.0002), and 93% (*P *= 0.0003), respectively. The most common adverse events were injection-site reactions (reported by 25%, 11%, 13%, and 57% of patients in the placebo and garadacimab 75-, 200-, and 600-mg groups, respectively). No serious adverse events, anaphylaxis, or thromboembolic events were reported. Phase 3 trials are ongoing.

Administration of an antisense oligonucleotide targeting plasma prekallikrein messenger RNA (Figure 2), which has been shown to prevent bradykinin formation (74), has been reported for 2 patients with severe bradykinin-mediated forms of HAE (75). The patients were initially treated with the parent antisense oligonucleotide IONIS-PKK_Rx_ SC for 12 to 16 weeks, followed by treatment with a ligand-conjugated form of the oligonucleotide, donidalorsen (formerly named IONIS-PKK-L_Rx_) 80 mg SC every 3 to 4 weeks for 7 to 8 months. IONIS-PKK_Rx_ SC dosing was 200 mg once weekly, with optional dose loading in the first 2 weeks. If the 2 patients had breakthrough HAE attacks, dosing could be increased to 300 mg after 6 weeks and to 400 mg after 12 weeks. During IONIS-PKK_Rx_ treatment, the mean monthly attack rate decreased from 1.2 to 0.2 attacks (patient 1) and from 7.9 to 1.0 attacks (patient 2). When patients were switched to donidalorsen (IONIS-PKK-L_Rx_), the mean monthly attack rate was 0 (patient 1) and 3.4 (patient 2). Plasma prekallikrein levels were reduced with both IONIS-PKK_Rx_ and donidalorsen (IONIS-PKK-L_Rx_) treatment. Both patients experienced injection-site reactions.

A phase 2, randomized, double-blind, placebo-controlled trial was conducted to further examine the efficacy and safety of once monthly SC donidalorsen 80 mg in adults with HAE (76). During the ≤8-week study run-in period, the 20 patients (donidalorsen [*n* = 14]; placebo [*n* = 6]) included in the trial experienced a mean 2.7 HAE attacks/month (range, 1.0 to 5.6) (76). Donidalorsen significantly reduced the mean number of monthly attacks compared with placebo (0.2 vs. 2.2, respectively; 90% difference; *P *< 0.001) (76). Donidalorsen decreased plasma prekallikrein activity from baseline by 61% (76). The most commonly reported adverse events with donidalorsen were headache (14%) and nausea (7%); no patients in either treatment group experienced serious adverse events or discontinued the trial due to adverse events (76).

## Discussion

Several options are available as on-demand treatment and as short-term or long-term prophylaxis of HAE attacks (2). Direct replacement with C1-INH products (e.g., pdC1-INH and rhC1-INH) treats all affected pathways, whereas single-target (pathway) therapies affect different components of the kallikrein-kinin system. Approved treatments for HAE vary in terms of indication(s), age limitations, route of administration, and frequency of dosing. C1-INH replacement therapies have a well-established efficacy and safety profile, given their long-term history of use in HAE (77), and newer agents are now being incorporated into HAE treatment regimens (78). Data for the C1-INH replacement therapies support dose-dependent efficacy, including the achievement of a zero-rate attack frequency (50, 54). Notably, the efficacy of C1-INH products for long-term prophylaxis was generally demonstrated in populations with a more severe HAE phenotype (on-placebo or pretreatment attack rate ∼4‒7 attacks/month) (40, 50, 54) than in similar trials of single-pathway treatment options (∼2–4 attacks/month) (Tables 3, 4) (63, 68). However, the phase 2 trial of garadacimab, an agent that targets both contact and fibrinolytic pathways, included patients with a more severe phenotype (mean 3.5–7.5 attacks/month) (73).

Although on-demand and prophylactic C1-INH treatments are effective for many patients with HAE (Tables 2–4), some patients may experience an increase in frequency of attacks while taking prophylactic therapy. Some authors have suggested that patients may develop tolerance to prophylactic therapy over time (52, 53). However, alternative explanations must be considered. For example, many patients lacking easy access to on-demand treatment do not treat every attack, and a percentage of attacks may remain untreated for other reasons (9, 79), so an increase in C1-INH usage may merely reflect better access to therapy and better patient education rather than tolerance induction. Prospectively collected observations based on patients receiving higher doses of prophylactic C1-INH do not suggest an increase in attack frequency and, if anything, show improved symptom control during 30 months of treatment, which disputes the theory of tolerance induction with C1-INH replacement therapy (51).

Long-term response to treatment may be dependent on baseline C1-INH levels in each patient (49). This is likely true for prophylaxis with C1-INH replacement therapies, but this hypothesis has not been specifically studied. Differences in study design, particularly in baseline or placebo HAE attack rates, treatment dosing, and route and frequency of administration, and a lack of head-to-head comparisons, make it difficult to evaluate any such relationship. For single-pathway therapies, genetic polymorphisms and perhaps environmental factors affecting other relevant pathways may be the most important predictors, along with factors affecting the degree of inhibition of the contact pathway target. Again, more research and better biomarkers are needed to determine the optimal approach for the individual patient. Given the lack of head-to-head clinical trials and differences among studies in patient populations, methods, and outcomes, which limit the ability to compare across trials, research is also needed to directly compare therapies in patients with HAE. Because both HAE therapies and a failure to control HAE symptoms have pharmacoeconomic implications (e.g., direct and indirect costs, including health care utilization), more data are needed about individual determinants of treatment response and the relative efficacy of HAE therapies (i.e., on-demand, short-term, and long-term prophylaxis) to enable delivery of an optimized treatment strategy for each patient.

In view of the wide variety of potentially relevant pathways regulated by C1-INH, it is perhaps surprising that several therapies that target only the contact pathway can produce a high level of control of angioedema (Figure 2). This reflects the central role of the contact pathway in angioedema generation and the multiple feedback loops between the contact pathway and other pathways implicated in angioedema generation. Further, new targeted agents in development for HAE have shown promise, albeit in small populations (73, 75).

In conclusion, some therapies for HAE target multiple pathways (i.e., C1-INH replacement therapy), while others target a single pathway (e.g., kallikrein inhibitors). Future research is needed to optimize therapeutic strategies and, in particular, to compare long-term outcomes both for angioedema control and for other consequences of C1-inhibitor deficiency, such as autoimmunity. In the meantime, health care providers and patients should establish an individualized management strategy that considers on-demand treatment and short-term prophylaxis, as well as long-term prophylaxis in appropriate patients, to minimize the disease burden of this condition.

## Contribution to the field statement

Hereditary angioedema (HAE) is a rare genetic disease that causes unpredictable, recurrent episodes of skin/mucosal swelling (called attacks) that can affect multiple locations, such as the hands, feet, gastrointestinal tract, other intra-abdominal organs, face, and upper respiratory tract. This condition substantially impairs patient-related quality of life and interferes with their ability to function at home, work or school, or socially. Management of HAE aims to treat or, preferably, prevent attacks, improving quality of life. Swelling during HAE attacks is mediated by bradykinin. Different pathways contribute to bradykinin generation. Almost all cases of HAE are caused by deficiency or dysfunction of C1-esterase inhibitor (C1-INH), which under normal conditions regulates different pathways to inhibit bradykinin production. C1-INH replacement therapy has been used for decades to treat or prevent HAE attacks. While complete disease control with C1-INH replacement is possible, some patients experience breakthrough attacks. More recently available agents target a single pathway—the contact pathway—to prevent bradykinin generation or block the effects of bradykinin. We review the mechanism of action, efficacy, and safety of approved therapies, as well as investigational agents; consider the therapeutic potential of single-pathway treatment options vs. agents with broader effects; and identify gaps in research.

## Author contributions

This work was developed from a poster presented at the 12th C1-Inhibitor Deficiency and Angioedema Workshop, June 3–6, 2021 (virtual). Both authors have contributed to the conception, development, drafting, and finalization of the manuscript. Both authors agree to be accountable for the content of the work. All authors contributed to the article and approved the submitted version.
